# New Advances in Diabetes Genetics

**DOI:** 10.3390/ijms24065591

**Published:** 2023-03-15

**Authors:** Maurizio Delvecchio

**Affiliations:** Metabolic Disorders and Genetics Unit, Giovanni XXIII Children’s Hospital, 70125 Bari, Italy; mdelvecchio75@gmail.com

Diabetes mellitus constitutes a heterogeneous group of disorders characterized by chronic hyperglycaemia. Genetic background plays a role to some extent in type 1 and type 2 diabetes, but it plays a key role in rare forms such as monogenic and syndromic diabetes. Genetic testing may unravel the predisposition to develop diabetes and its consequences, and thus it provides robust evidence for clinical trials and personalized treatment. The molecular diagnosis is fundamental in rare forms to choose the appropriate treatment, to reduce the risk of consequences, and for genetic counselling. This Special Issue contains two reviews [1,2], three original papers [3,4,5], and one case report [6] that address genetic issues in diabetes mellitus.

Liguori et al. [1] present a comprehensive review about the genetics of diabetes with a focus on how the *Drosophila* model may contribute to the advancement of knowledge on diabetes. *Drosophila* is an interesting model to unravel the mechanisms underlying the impairment of insulin secretion and/or action. This fly is a reliable system to perform genetic screening, evaluate the progression of the disease and investigate its modifiers.

Sousa et al. [2] review the genetics and signalling pathways of MODY. Their paper covers the mechanisms underlying hyperglycaemia and provides comprehensive and interesting insights for the readers. This rare form of diabetes is an interesting model for personalized medicine [2,7].

Wolfram syndrome is a rare neurodegenerative disorder, which may present diabetes mellitus, optic atrophy (key elements of this syndrome), diabetes insipidus, and deafness. Here, we present an adolescent carrying a segmental uniparental disomy encompassing the novel maternally inherited c.1369A > G; p.Arg457Gly variant [3]. We have previously showed that, up to date, the pathogenic variant does not predict the phenotype so far [8], and thus expanding the spectrum of genetic variants may be useful.

Several loci are involved in type 2 diabetes mechanisms. Tibori et al. [4] focused their attention on the effect of the missense rs2234970 single-nucleotide polymorphism (SNP) on stearoyl-CoA desaturase-1 activity. This enzyme plays an important role in the synthesis of unsaturated fatty acids. In their in vitro study, this SNP contributes to the onset of obesity-related metabolic disorders, such as type 2 diabetes. Expanding the knowledge of the genetic backgrounds of insulin action impairment can help to prevent disease onset in patients prone to developing such disorders.

Similarly, Massarenti et al. [5] investigated the role of some SNPs of insulin and insulin receptor genes in the synthesis of anti-insulin antibodies. Interestingly, they show that the rs3842752 and the rs689 alleles in the insulin gene may reduce the risk of anti-insulin antibodies synthesis, while the rs2245649 and rs2229429 alleles in the insulin receptor gene are associated with poor glycaemic control.

The DIAMATER study cohort is a prospective cohort recruited to investigate the biomolecular muscle profile as predictor of long-term urinary incontinence in women with gestational diabetes mellitus (GDM). In this Special Issue, Alves et al. [6] describe the transcriptome profiling of the rectus abdominis muscle obtained via caesarean section from pregnant women with or without GDM and with or without pregnancy-specific urinary incontinence. They describe 650 genes showing different levels of expression, providing evidence for further studies to develop innovative and targeted strategies to prevent and to treat complications such complication.
